# Invasive micropapillary carcinoma of the breast and invasive breast carcinoma of no special type: a comparison of claudin proteins’ expression and its impact on survival

**DOI:** 10.3389/pore.2024.1611987

**Published:** 2024-12-02

**Authors:** Zsófia Kramer, András Budai, Adrián Pesti, Janina Kulka, Anna-Mária Tőkés

**Affiliations:** Department of Pathology, Forensic and Insurance Medicine, Semmelweis University, Budapest, Hungary

**Keywords:** invasive micropapillary carcinoma of the breast, invasive breast carcinoma of no special type, claudin expression, tight junction, prognosis

## Abstract

Invasive micropapillary carcinoma of the breast is characterized by clusters of cells presenting with inverted polarity. Although the apico–basal polarity is a fundamental property of the epithelium, the biological alterations leading to the inside-out pattern observed in invasive micropapillary carcinoma (IMPC) remain mostly unknown. The regulation of tight junctions in polarity formation and maintenance is acknowledged. By using immunohistochemistry, we have analysed claudin-1, -3, -4, and -7 tight junction proteins expression and their prognostic value on IMPCs and compared them to invasive breast carcinomas of no special type (IBC-NST) tumors. Our cohort consisted of 37 IMPCs, 36 IBC-NST and 9 mixed IMPC/IBC-NST tumors. Two scoring systems were used to quantify protein expression: a 4-tier scoring system and the H-score method. Distant metastasis free survival (DMFS) intervals and overal survival (OS) data were used for prognosis evaluation. The analysed samples were characterized mainly by low or no claudin-1 expression whereas claudins-3, -4 and -7 showed variable positivity. We have found no significant differences in claudin-3 and -4 protein expression between IMPC and IBC-NST groups with either scoring methods, however high claudin-7 expression was found in significantly more IMPCs than IBC-NST tumors according to the H-score system (*p* = 0.02). The 4-tier scoring method revealed association of claudin-7 expression with molecular tumor subtypes (*p* = 0.001). IMPC and IBC-NST tumors did not show difference in DMFS (*p* = 0.70). In the analysis of pure IMPC and IBC-NST tumors, positive/high claudin-4 protein expression was significantly associated with shorter DMFS (*p* = 0.02/*p* = 0.008, respectively according to the two scoring methods). Claudin-3 and claudin-7 expression showed no association with DMFS or OS. Changes in epithelial polarity seem not to be related to claudin-1, -3, and -4 expression. Increased claudin-4 expression may have a role in breast cancer progression.

## Introduction

Invasive micropapillary carcinoma of the breast, is a special carcinoma phenotype composed of small, hollow, or morula-like clusters of malignant cells, surrounded by clear spaces and presenting an inside-out growth pattern with epithelial membrane antigen (EMA). In pure invasive micropapillary carcinoma (IMPC), >90% of the tumour consists of hollow or morula-like aggregates of cuboidal to columnar neoplastic cells. IMPC has been described in tumors of several tissues, such as breast, urinary bladder, stomach, colon, pancreas and lung [1–4]. Although this histological subtype is well defined, the underlying mechanisms leading to its unique appearance are not fully understood. IMPCs comprise 1%–8.4% of all breast carcinomas [5, 6] and were previously thought to have poor prognosis, however, recent studies, as well as our previous study have shown no difference in outcome compared to invasive breast carcinomas of no special type (IBC-NST) [7–10].

Several research groups have studied the genetic alterations present in pure IMPCs and have found that these tumors comprise a heterogenous group with genetic alterations different from that of IBC-NST tumors [11, 12].

Cell polarity alterations and changes in the expression of cell adhesion and tight junction molecules have been widely studied in carcinogenesis and cancer progression, however their implication in the inside-out pattern observed in IMPCs is scarcely known. The majority of the studies focus on the eventual higher metastatic potential of IMPCs [13–15]. There are several open questions about the role of abnormal polarity in the direction of secretion and the interaction of these cells with tumor microenvironment.

Tight junctions, which form stable selective paracellular barriers between epithelial cells, are mostly located in the apical end of the lateral membrane of the cells, maintaining cell polarity and cell adhesion. Claudins, first described by Furuse et al. [16], are main components of the tight junctions and compose continuous strands in the apical region, but are also found along the lateral membrane as free strand ends. The turnover of claudins is continuous along the lateral membrane [17], providing stability to the tight junctions.

Claudins, together with other tight junction molecules play an important role in cell adhesion and cell polarity maintenance [18]. Zonula occludens-1, -2 and -3 (ZO-1, ZO-2, ZO-3) proteins independently, while junctional adhesion molecule A (JAM-A) together with claudins are required for epithelial polarity [19], among several other structures. The role of claudins in cancer progression have been in the focus of several studies. Altered expression of claudins in different cancer types plays role in tumor progression in a tissue-specific manner [20].

Of the 27 human claudins known to date the most intensively studied claudins in breast carcinomas are claudin-1, -3, -4 and -7. Several tumor features are associated with different claudins expression in breast carcinomas. Decreased or loss of claudin-1 expression has been shown to be associated with higher recurrence rate and metastatic potential and with poor prognosis [21]. On the other hand, high claudin-1 and -4 expression was found in a majority of “basal-like,” triple negative breast carcinomas [22–24]. Some studies have shown correlation between claudin-3 and -4 expression and tumor grade [22, 25, 26], while other studies have found that certain breast cancer subtypes are associated with different claudin expression levels with different prognostic significance [21–32]. High level of cytoplasmic claudin-3 expression in triple negative breast carcinomas has been associated with poor survival [28].

Increased claudin-4 expression has been associated with higher tumor grade and with basal like phenotype [24, 32]. Decreased/loss of claudin-7 expression has been shown to correlate with histological grade in DCIS lesions as well as in invasive carcinomas [29, 31].

A subset of breast carcinomas show claudin “low” expression profile (as defined by decreased gene-expression of claudins-1, -3, -4, -7, and -8 [23], or by decreased expression of claudin-3, -4, -7, E-cadherin and calcium-dependent cell-cell adhesion glycoprotein [27, 30]). Histologically these tumors are mostly triple negative, high-grade tumors, commonly showing metaplastic or medullary features [23, 27, 30].

In our previous study we have compared claudin expression profiles of IMPCs and IBC-NST tumors on RNA level. We have found higher expression levels of *CLDN*s 3, 4, and 7, and lower *CLDN1* levels in IMPCs. We have also shown that high *CLDN3* expression level is associated with grade 3 tumors and with worse distant metastasis free survival (DMFS) [10].

The aim of our current study was to examine claudin expression on protein level in a mostly similar cohort, to analyse whether their expression is associated with prognosis and to compare RNA and protein expression levels of claudins.

To date – to the best of our knowledge - claudin protein expression has not been examined in IMPCs. Claudins, as part of the tight junction proteins might play role in the special histological appearance of IMPCs. Understanding their role may open new possibilities for targeted therapy of this special tumor subtype.

## Materials and methods

Eighty-two breast carcinoma cases [37 IMPC, 36 age- and stage-matched IBC-NST (for statistical comparison) and 9 mixed IMPC/IBC-NST cases] were selected from the archive of the Department of Pathology, Forensic and Insurance Medicine - Semmelweis University, Budapest from the period of 2000–2021, with the ethical permission of Semmelweis University Research Ethics Committee (permission number: 240/2016). All cases were reviewed by expert pathologists and classified based on the World Health Organisation criteria [33]. Additionally, IMPC cases were confirmed by the typical inside-out staining pattern of EMA immunohistochemistry [34] (performed with automated Ventana BenchMark ULTRA system using Cell Marque Mouse Monoclonal antibody, 1:200). Breast cancer surrogate molecular subtypes were defined according to the St. Gallen International Expert Consensus – 2013 [35]. Tumor characteristics and patient data as well as clinical follow up information were obtained from the Semmelweis University Health Care Database and the National Cancer Registry.

### Immunohistochemical analysis of claudin-1, -3, -4, and -7

Formalin fixed paraffin embedded tissues were used to perform immunohistochemical studies. 3–5 μm thick sections were cut, and immunohistochemical reactions were performed on Ventana BenchMark Ultra system according to the Universal UltraView DAB manufacturer’s protocol. The following primary antibodies were used: claudin-1 (Cell Marque, Rabbit polyclonal antibody, 1: 100), claudin-3 (Invitrogen, Rabbit polyclonal antibody, 1:100), claudin-4 (Invitrogen, Rabbit polyclonal antibody, 1:100), claudin-7 (Invitrogen, Rabbit polyclonal antibody, 1:100). All primary antibodies were incubated for 32 min on 42°C. Counterstaining with haematoxylin was used after antibody visualisation. All immunohistochemical reactions were performed using external positive control tissue.

### Quantification of claudin expression

Slides were scanned with 3D HISTECH Pannoramic^®^ 1000 digital slide scanner. All immunohistochemical slides were analysed by one expert histopathologist (ZK) on digitized slides. Twenty percent of the cases were analysed by a second expert (AT), the two results were concordant. In cases of mixed IMPC/IBC-NST tumors the two components were separately evaluated. Accordingly 91 samples, 46 IMPC and 45 IBC-NST were analysed.

No standardized methods are available for the quantification of claudin proteins expression [25, 36, 37].

Two methods were used to quantify the IHC results:

a. A 4-tier immunohistochemical score system was applied on the cohort. No evidence of membranous or cytoplasmic staining was evaluated as score 0, increasing staining intensities were scored from 1+ to 3+. Samples showing score 0 were declared as negative, score 1+, 2+ and 3+ were grouped as positive samples for protein expression.
b. H-score was determined by adding the results of multiplication of the percentage of cells with staining intensity ordinal value (scored from 0 for “no signal” to 3 for “strong signal”) with values between 0 and 300. Low and high expression was determined by calculating median values. H-score values above the median were considered as high expression and those below the median were considered low expression.

### Statistical analysis

Clinical data and histology score values were processed using JupyterLab with R language (v 4.2.0). Homogeneity test of data was performed by Kolmogorov-Smirnov test. Categorical data were compared using Chi-square test. Cox proportional hazard model calculation was performed to evaluate predictive and hazard value of variables, both regarding DMFS and OS. The results of this calculation were indicated as hazard ratio (HR) and confidence intervals (CI). *P*-values presented in the tables are representative to population wise measurements (compared to the reference variable or between positive and negative expression) and are not subgroup (IBC-NST, IMPC or Mixed IMPC/IBC-NST) related due to statistical adequacy. Mixed IMPC/IBC-NST cases were excluded from survival analysis due to the low patient number. Plotting was performed using ggpubr and survminer package. *P*-values <0.05 were considered statistically significant. Survival curves calculated by Cox proportional hazard model were created with ggsurvplot function of ggplot2 R package.

## Results

### Patient characteristics

Tumors of 82 breast cancer patients were included in our study, 36 IBC-NST, 37 IMPC and 9 mixed IMPC/IBC-NST tumors were examined. Mixed tumor components were analysed separately for protein expression and were included to the IMPC (46 samples) and IBC-NST (45 samples) groups respectively (totally 91 samples). Median age of the patients was 63 years in the IBC-NST group, 63 years in the IMPC and 61 in the mixed IMPC/IBC-NST group. About half of the patients presented with lymph node metastasis (42/82), and most of the tumors were grade 2, stage pT1-2. Median follow up time was 49 months (range: 0–198 months). Distant metastasis occurred in 25 out of 82 cases (14/36 in IBC-NST, 8/37 in IMPC and 3/9 in mixed IMPC/IBC-NST cases).

Patients’ and tumors’ characteristics are shown in Table 1. All three patient groups showed similar distribution regarding age and prognostic factors.

**TABLE 1:** Patient characteristics.

	IBC-NST	IMPC	Mixed IMPC/IBC-NST	*p*-value*
Total patient number	36	37	9	
Number of samples examined	45	46		
Median years of age (range)	63 (34–83)	63 (33–85)	61 (34–69)	
Median of Ki67 LI (range)	15 (1–100)	15 (1–90)	16 (5–90)	0.22^a^
Grade I II III	3 (8.3%) 20 (55.5%) 13 (35.2%)	3 (8.1%) 23 (62.2%) 11 (29.7%)	1 (11.1%) 4 (44.45%) 4 (44.45%)	0.90^b^
T 1 2 3 4	14 (38.9%) 11 (30.6%) 8 (22.2%) 3 (8.3%)	18 (48.6%) 8 (21.6%) 8 (21.6%) 3 (8.1%)	3 (33.3%) 4 (44.5%) 1 (11.1%) 1 (11.1%)	0.85^b^
N 0 1 2 3	17 (47.2%) 8 (22.2%) 6 (16.7%) 5 (13.9%)	20 (54.1%) 8 (21.6%) 3 (8.1%) 6 (16.2%)	3 (33.3%) 4 (44.5%) 1 (11.1%) 1 (11.1%)	0.73^b^
ER + -	27 (75%) 9 (25%)	35 (94.6%) 2 (5.4%)	8 (88.9%) 1 (11.1%)	0.05^b^
PR + −	17 (47.2%) 19 (52.8%)	29 (78.4%) 8 (21.6%)	8 (88.9%) 1 (11.1%)	0.005^b^
HER2 + −	5 (13.9%) 31 (86.1%)	8 (21.6%) 29 (78.4%)	1 (11.1%) 8 (88.9%)	0.59^b^
Distant metastasis Absent Present	22 (61.1%) 14 (38.9%)	29 (78.4%) 8 (21.6%)	6 (66.7%) 3 (33.3%)	0.27^b^
Surrogate molecular subtypes LUM-A LUM-B1 LUM-B2 HER2 positive TNBC	10 (27.8%) 14 (38.9%) 3 (8.3%) 2 (5.6%) 7 (19.4%)	20 (54.1%) 7 (18.9%) 8 (21.6%) 0 2 (5.4%)	4 (44.5%) 3 (33.3%) 1 (11.1%) 0 1 (11.1%)	0.93^b^

^a^ Kruskal-Wallis test.

^b^ Chi-square test.

IMPC, invasive micropapillary carcinoma; IBC-NST, Invasive breast carcinoma - no special type; ER, estrogen receptor; PR, progesterone receptor; LI, labeling index (%); LUM-A, Luminal-A type breast carcinoma; LUM-B1, Luminal B-HER2 negative type breast carcinoma; LUM-B2, Luminal B-HER2 positive type breast carcinoma; TNBC, triple negative breast cancer.

### Localization and expression of the analysed proteins

**FIGURE 1:**
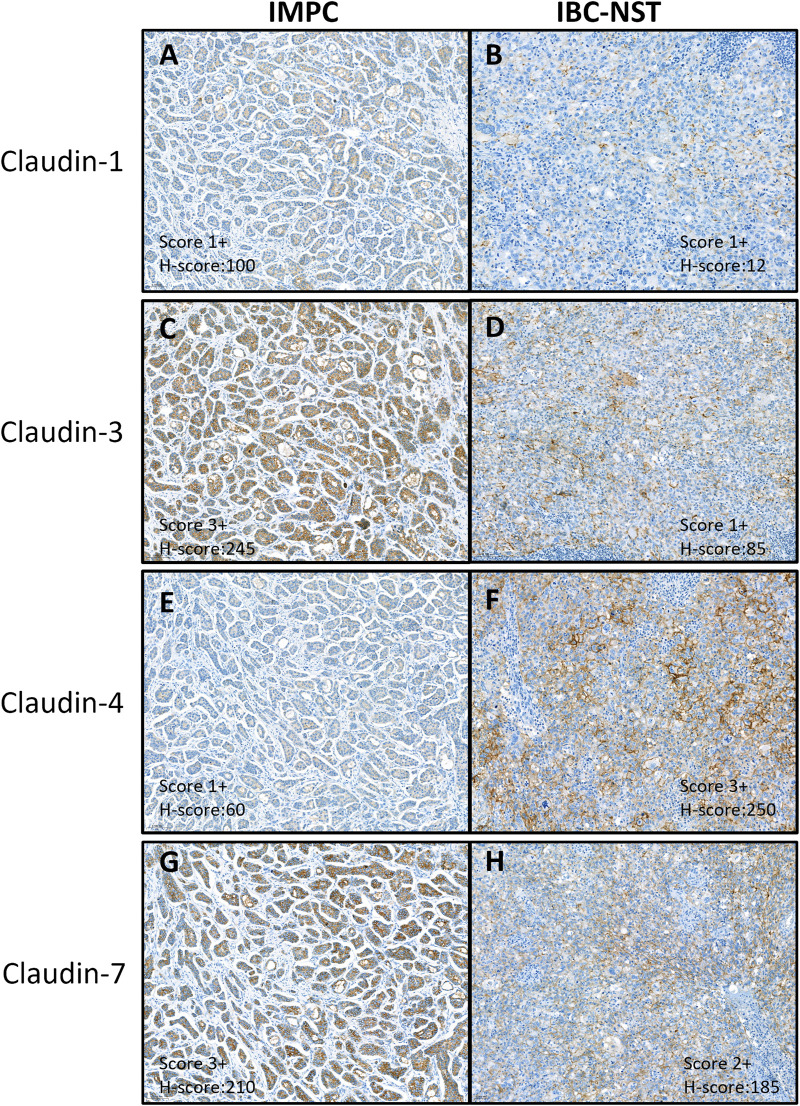
Immunohistochemical expression of claudin proteins. Immunohistochemical analysis showed mainly weak or no cytoplasmic and/or membrane staining of claudin-1 **(A, B)** and variable staining intensity of claudin-3 **(C, D)**, −4 **(E, F)**, and −7 **(G, H)**. **(A, C, E, G)** is a sample of IMPC, grade 2, LUM-A tumor. **(B, D, F, H)** is a sample of IBC-NST, grade 3, TNBC tumor. IMPC, Invasive micropapillary carcinoma; IBC-NST, Invasive breast carcinoma - no special type; LUM-A, Luminal-A type breast carcinoma; TNBC, Triple negative breast cancer.

Protein expression of claudin-1 was mainly weak membranous and/or cytoplasmic or negative by immunohistochemical staining. Claudins-3, -4 and -7 showed variable intensity, mostly circumferential or partial membrane positivity (Figure 1).

### Semiquantitative scoring method results

After scoring our cohort according to the traditional 0, 1+, 2+, 3+ system, we have found no significant difference between claudin-1, -3, -4, and -7 protein expression levels between the IMPC and IBC-NST groups (*p* = 0.17, 0.31, 0.34, 0.22, respectively).

None of the samples (0/91) showed high claudin-1 expression (score 3+) whereas high claudin-3 expression (score 3+) was detected in 5/91 samples (2 IBC-NST, 3 IMPC), high claudin-4 in 1/91 sample (IBC-NST) and high claudin-7 expression was seen in 13/91 samples (5 IBC-NST and 8 IMPC).

8/91 samples (6 IBC-NST, 2 IMPC) were negative for claudin-3, -4 and -7 whereas 36/91 samples (15 IBC-NST, 21 IMPC) presented with positivity for all of the above three claudins.

Cytoplasmic positivity was also seen in a minority of samples (8.8%, 32/364) in both IMPC and IBC-NST groups (11 and 21, respectively) as follows: 9.9% (9/91) of claudin-1, 8.8% (8/91) of claudin-3, 12% (11/91) of claudin-4, and 4.4% (4/91) of claudin-7.

### H-score analysis

To compare the proteins expression with mRNA analysed in our earlier study [10] the results of H-score evaluation was applied. Variable staining intensity of claudins-1, -3, -4 and -7 was observed. Median H-score values of claudin-3 and claudin-7 expression were 115 and 150, respectively. Most of our samples showed no staining with claudin-1 (median value 0.5). Claudin-1 H-score above 100 occurred in 5 samples, showing no correlation with tumor subtype, grade, or receptor status. Claudin-4 expression was below 50 to no expression in 60/91 of the samples, with a median value of 10. Expression values between 50 and 100 of claudin-4 was found in 14 samples. Claudin-4 values above 100 were found in 17 samples, with no connection to tumor characteristics.

### Comparison of protein and RNA expression

In our previous study [10] we have examined mRNA expression on a largely identical cohort to this present study by using NanoString nCounter Analysis system. Median values of mRNA expression levels were used as threshold for determining low and high expression for each of the examined 43 genes. We have shown, that *CLDN3*, *CLDN4* and *CLDN7* mRNA expression is significantly higher in IMPC tumors compared to the IBC-NST group, while *CLDN1* showed significantly lower mRNA expression in the IMPC group. Examining protein expression of claudin-1, -3, and -4 did not show differences in the two histological groups. We have compared mRNA expression levels of our previous study and H-score values of the current study. Median values were very low for both *CLDN1* mRNA (184.74) and claudin-1 protein expression (0.5). Interestingly median value of claudin-4 protein expression was low [10], compared to the high median value of *CLDN4* mRNA expression (3683.8). High mRNA expression correlated with high protein expression in about 2/3 of samples (claudin-3: 68%, claudin-4: 61% and claudin-7: 70%), while samples showing low mRNA expression have also shown low protein expression in 36% (claudin-3), 49% (claudin-4) and 34% (claudin-7) of the samples. In case of claudin-1, the correlation was 52% (high expression) and 47% (low expression).

### Breast cancer subtype distribution

Of the analysed cases 94.6% of the IMPCs and 75% of IBC-NST samples were hormone receptor positive (HR+). Analysing separately the HR+ and HR- samples we have found that the distribution of the different claudins in the HR+ samples was the following: 56.71% were claudin-3 positive, 46.1% claudin-4 positive and 89% claudin-7 positive. In the HR- samples this ratio was 69%, 53% and 53%, respectively.

The distribution of the different claudins in each molecular and histological subtype is presented in Table 2 (after evaluation according to the 4-tier method) and Table 3 (after evaluation according to the H-score). The most representative subtype in our cohort was the LUM-A subtype (38/91, 41.7%) characterized by claudin-3 and claudin-7 positivity and claudin-4 negativity in 76%, 92% and 58% of the samples, respectively. The 4-tier evaluation showed that claudin-7 expression is associated with molecular subtype distribution (*p* = 0.001). Evaluation of the samples with the H-score method showed a mostly similar distribution of histological and molecular subtypes in cases of claudin-3 and claudin-4 expression. According to the histological subtype, high claudin-7 expression occurred in significantly more IMPC cases compared to IBC-NST tumors (*p* = 0.02).

**TABLE 2:** Claudin expression distribution between molecular subtypes in the 91 samples using the 4-tier method.

	Claudin-1 neg.	Claudin-1 pos.	*p*-value	Claudin-3 neg.	Claudin-3 pos.	*p*-value	Claudin-4 neg.	Claudin-4 pos.	*p*-value	Claudin-7 neg.	Claudin-7 pos.	*p*-value
All	76	15		26	65		48	43		14	77	
IBC-NST	40	5	0.17^a^	15	30	0.31^a^	26	19	0.34^a^	9	36	0.22^a^
IMPC	36	10		11	35		22	24		5	41	
LUM-A	29	9	0.76^a^	9	29	0.59^a^	22	16	0.67^a^	3	35	0.001^a^
LUM-B1	27	0		10	17		12	15		4	23	
LUM-B2	9	4		3	10		8	5		1	12	
HER2	2	0		0	2		0	2		0	2	
TNBC	9	2		4	7		6	5		6	5	

^a^ Chi square test.

IMPC, invasive micropapillary carcinoma; IBC-NST, Invasive breast carcinoma - no special type; LUM-A, Luminal-A type breast carcinoma; LUM-B1, Luminal B-HER2 negative type breast carcinoma; LUM-B2, Luminal B-HER2 positive type breast carcinoma; TNBC: triple negative breast cancer.

**TABLE 3:** Claudin expression distribution between molecular subtypes in the 91 samples using the H-score method.

	Claudin-3 low	Claudin-3 high	*p*-value	Claudin-4 low	Claudin-4 high	*p*-value	Claudin-7 low	Claudin-7 high	*p*-value
All	44	47		41	50		44	47	
IBC-NST	24	21	0.34^a^	20	25	0.90^a^	27	18	0.02^a^
IMPC	20	26		21	25		17	29	
LUM-A	22	16	0.47^a^	19	19	0.69^a^	18	20	0.76^a^
LUM-B1	13	14		10	17		11	16	
LUM-B2	5	8		7	6		7	6	
HER2	0	2		0	2		1	1	
TNBC	4	7		5	6		7	4	

^a^ Chi square test.

In our cohort 8 samples were considered as negative for claudin-3, -4, and -7 with immunohistochemistry (IHC) (claudin all low group). Four samples were LUM-A, 2 samples LUM-B1 and 2 samples TNBC subtype. Due to the relatively low number of samples, further statistical analysis was not performed on the claudin all low group. Of the 45 IBC-NST samples, 6 were negative for claudin-3, -4, and -7 whereas of the 46 IMPC samples 2 were negative for claudin-3, -4, and -7.

### Analysing the prognostic value of claudin-3, -4, and -7 expression

**FIGURE 2:**
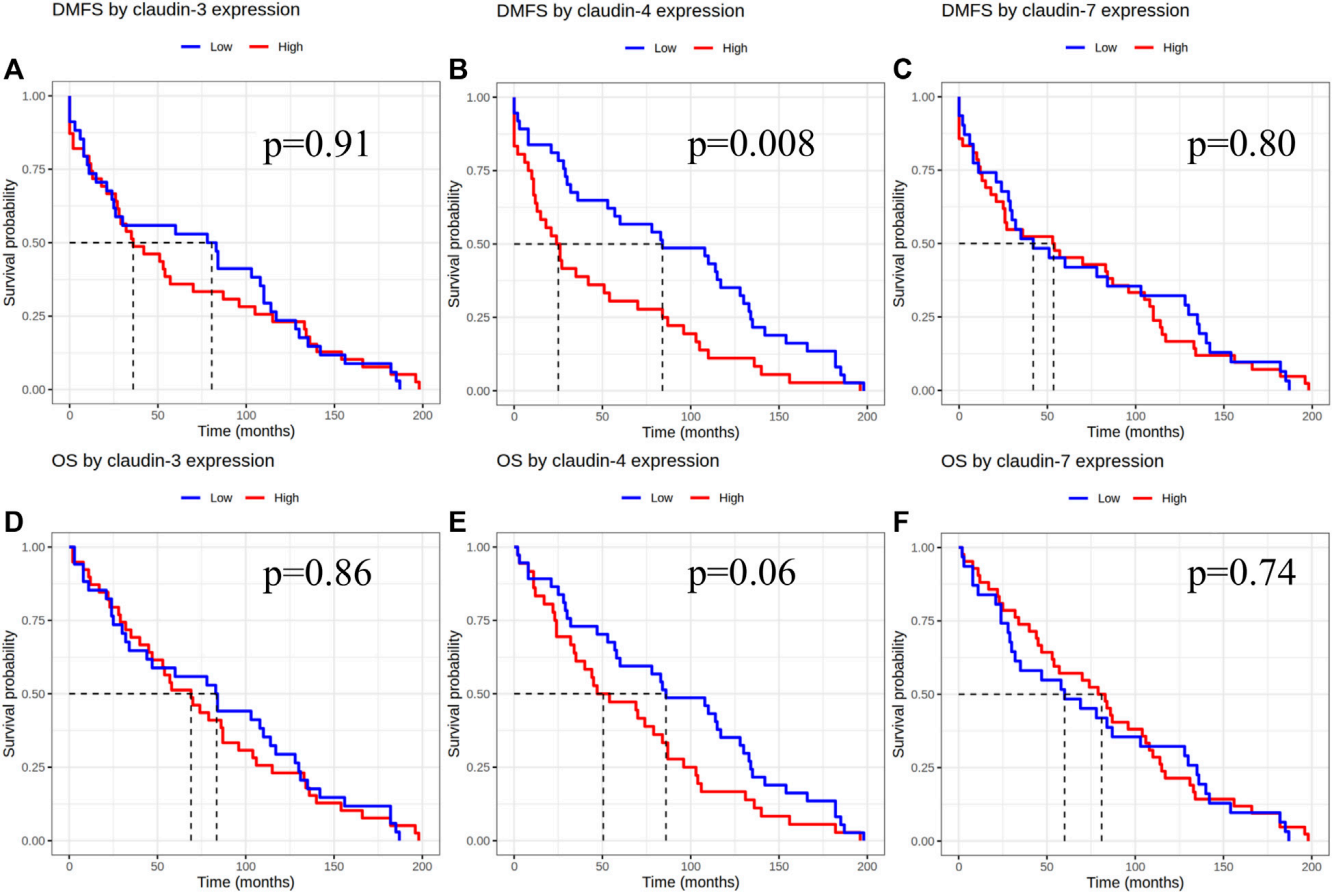
Claudin expression effect on DMFS and OS after evaluation of immunoexpression according to the H-score. DMFS by claudin-3, -4 and -7 expression **(A–C)**, OS by claudin-3, -4 and -7 expression **(D–F)**. DMFS, Distant metastasis free survival; OS, Overall survival.

Statistical analysis was performed with both scoring methods. Based on H-score evaluation claudin-1 staining showed low to no reaction, with low median values (0.5), low claudin-4 expression was detected in 45% of the samples, and the median value of claudin-4 expression was low (H-score 10). Due to the extremely low median value of claudin-1, we did not perform further statistical analysis on claudin-1 expression. Low claudin-4 expression was associated with significantly longer DMFS [*p* = 0.008 (HR-CI): 0.529 (0.329–0.848)] when examining the pure IMPC and IBC-NST cases. Claudin-3 and claudin-7 expression did not show any correlation with DMFS [*p*-values 0.91 (HR-CI): 0.976 (0.611–1.557) and 0.80 (HR-CI): 0.943 (0.588–1.510), respectively]. Claudin expression did not show correlation with overall survival (Figure 2).

**FIGURE 3:**
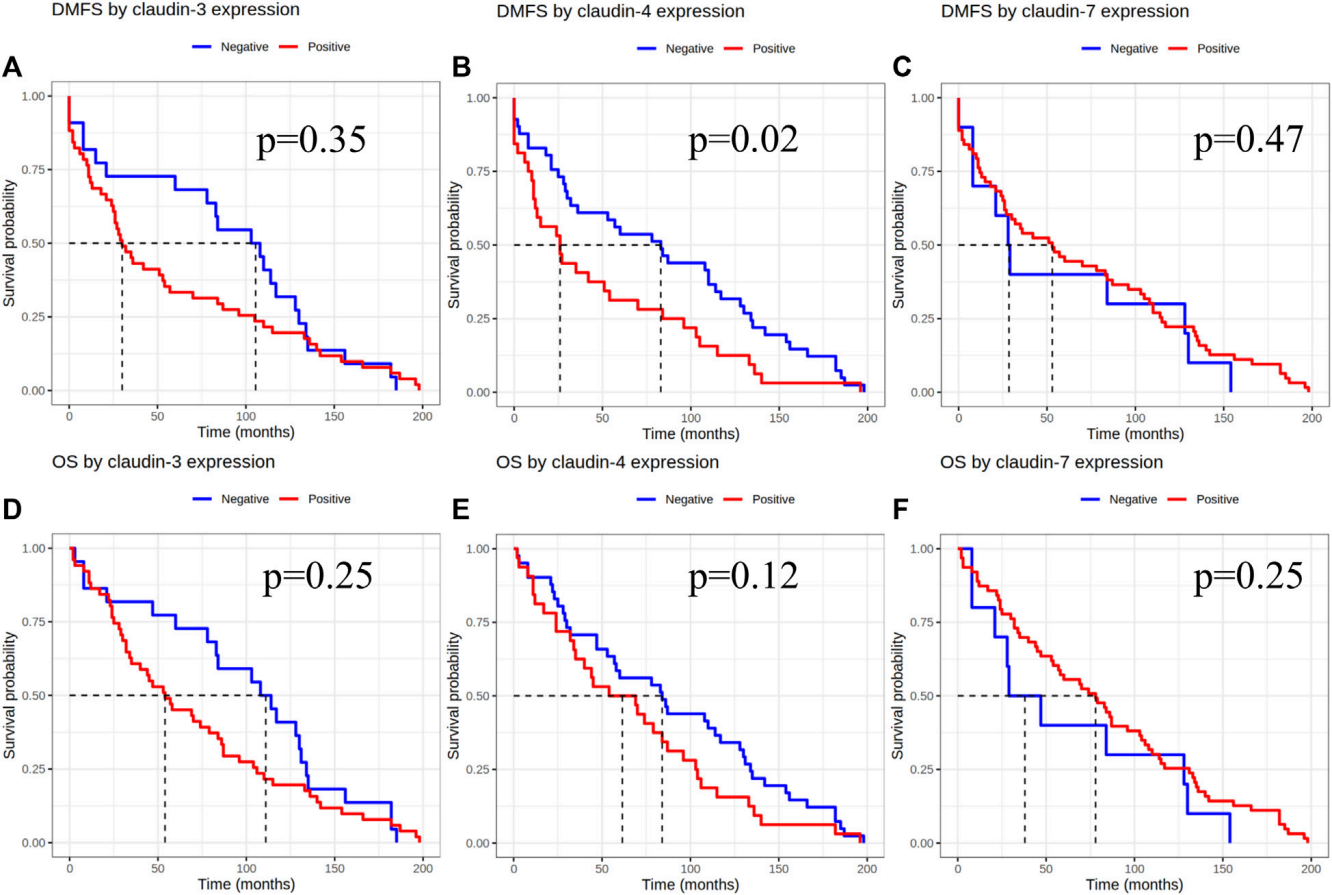
Claudin expression effect on DMFS and OS after evaluation of immunoexpression using the 4-tier system. DMFS by claudin-3, -4 and -7 expression **(A–C)**, OS by claudin-3, -4 and -7 expression **(D–F)**. DMFS, Distant metastasis free survival; OS, Overall survival.

Based on the results of the 4-tier and examining pure IMPC and IBC-NST cases, samples considered as claudin-4 negative by IHC were also associated with significantly longer DMFS [*p* = 0.02, HR-CI: 1.744 (1.084–2.804)], but no difference was seen in overall survival [*p* = 0.12, HR-CI: 1.446 (0.903–2.315)]. Claudin-3 and -7 expression did not show any effect on overall survival or DMFS (Figure 3).

## Discussion

In the present study, 45 IBC-NST and 46 IMPC samples (36 IBC-NST, 37 IMPCs and 9 mixed IMPC/IBC-NST cases with 91 tumor samples) were evaluated for claudin-1, -3, -4, and -7 immunohistochemical expression in search for the possible role of these markers in the formation of inverted polarity, in differentiating between IBC-NST and IMPC subtypes and as potential prognostic indicators. To date no standardized methods are available for the quantification of claudin proteins expression. Some study groups use a semiquantitative scale from 0 to 3, others the H-score, while there are studies presenting the results of a unique scoring system by combining staining intensity and percentage of positive cells. Not just the methods differ between study groups, but also the cut-off values to distinguish positive and negative cases are different [25, 36–42].

In one of our earlier studies by analysing fresh and FFPE breast tissues we demonstrated that claudin-1 protein is absent, or its expression is markedly decreased in the majority of different types of invasive breast carcinomas as compared with normal ducts and *CLDN1* mRNA expression investigated by real-time PCR confirmed this finding. Related to claudin-4 our earlier study showed downregulated claudin-4 protein expression in grade 1 IDC-NSTs [32]. In this current cohort claudin-4 downregulation was not correlated with tumor grade, however positive claudin-4 expression was associated with a shorter DMFS.

IMPC tumors have a distinct histological appearance. As we have shown in our previous study, this subtype shows higher mRNA expression levels of *CLDN*s 3, 4, and 7, and lower *CLDN1* levels, when compared with IBC-NST tumors, which might contribute to the unique histological features [10]. In our current, mostly similar cohort, we could not show differences between the two groups on the protein expression level. Correlation between gene and protein expression of claudins has been studied by Li et al. [43]. They have shown that the expression levels do not necessarily correlate with each other, which can occur due to epigenetic alterations, transcription factors, RNA alternative- or mis-splicing, posttranslational modifications, or signalling pathway effects. In our study we have also seen only partial correlation between RNA and protein expression levels.

Polarity formation and maintenance require the regulation of tight junctions and accordingly the involvement of the actin and microtubule cytoskeleton. Although several proteins participate in maintaining apicobasal polarity, three major polarity complexes are mentioned as the core proteins. These are the apical Crumbs and Par complexes and the basolateral Scribble complex [44–46].

It is known that tight junction proteins modulate various signalling cascades which have effect on cellular differentiation, growth, proliferation and cell migration. Polarity switching has been described as a critical step in metastasis formation. Tumor cell clusters may switch from apical-in to apical-out polarity in the course of vascular invasion. Integrins are key molecules in the interaction between the cells and the extracellular matrix. Intracellular activation of cytoskeletal and regulatory proteins by integrin signalling has been described to be activated in cancer [47]. Several signalling pathways have been linked to tight junction proteins, including TGF-ß-dependent pathway signalling, Ras-Raf-MEK-ERK and PI3K/Akt signalling, Wnt/ß-catenin signalling, STAT signalling, the Hedgehog and the Notch pathways [48].

Based on our results changes in epithelial polarity in IMPCs seems not to be related to claudin-1, -3, and -4 expression as the distribution of the mentioned proteins was mostly similar in IMPC and IBC-NST tumors. However high claudin-7 expression occurred in significantly more IMPC cases compared to IBC-NST tumors.

Tetsuhisa et al. tested the role of TJs in epithelial polarity by systematically knocking out TJ components and they have found that epithelial polarity was disorganized in ZO-1/ZO-2–deficient cells, but not in claudin-deficient cells. They concluded that claudins and JAM-A co-ordinately regulate TJ formation and epithelial polarity [19].

The prognostic role of different claudins have been analysed in several studies. Changes in claudin expression have different effects in different cancer types. In breast cancer claudin-4 overexpression has been associated with progression, migration and worst prognosis, which is in concordance with our findings [25, 26, 41, 49]. In triple negative breast carcinomas (TNBC) high cytoplasmic but not membranous claudin-3 and claudin-7 expression is predictive of poor outcome according to a study group [28], while another group has shown that membranous claudin-3 overexpression is associated with poor survival in TNBCs [26]. Claudin-7 expression was shown to be lower in high grade breast carcinomas and decreased expression was found in ER- tumors [29, 50].

Claudin low phenotype of breast carcinoma is defined by low expression of cell adhesion genes, high expression of epithelial-mesenchymal transition genes, and stem cell-like expression pattern [30]. These tumors show marked immune and stromal cell infiltration, low level of genomic instability and lower proliferation rates. Fougner et al. have re-evaluated the characteristics of claudin low breast cancer subtype tumors and have found that these tumors are not a distinct, sixth subtype of breast carcinomas. Claudin low tumors are found in all breast cancer subtypes, showing characteristics closer to their intrinsic subtype rather than to claudin low tumors [51]. Claudin low tumors show low gene expression levels of claudins-3-, -4, and -7.

It would be interesting to analyse whether the loss of several claudins is associated with inversed cell polarity. In our cohort only a low number of samples were considered as negative by IHC for all three claudins (6 IBC-NST and 2 IMPC) so, further analysis was not performed on this group. A recent study has described that the decrease or loss of claudin expression is accompanied by cell-cell adhesion- and polarity damage [52].

Molecular therapies that target claudin-4 are being developed continuously. As seen in many studies [25, 26, 41, 49, 53], including our cohort, high claudin-4 expression is associated with worse prognosis. Luo Yi et al. showed that anti-claudin-4 extracellular domain antibody, 4D3 enhances the chemotherapeutic antitumor effect of paclitaxel in two human breast cancer cell lines [53]. Patients with breast carcinomas showing high claudin-4 protein expression may benefit from anti-claudin-4 antibody treatment as part of the treatment protocol. Hence, the specific antibody connection enhancing the chemotherapeutic effect in these potentially high-risk tumors may increase patient survival.

## Conclusion

Changes in epithelial polarity seems not to be related to claudin-1, -3, and -4 expression as IMPC and IBC-NST tumors showed mostly similar expression of these proteins in our cohort. Differences were observed in claudin-7 protein expression between the two histological subtypes. No statistically significant differences were detected in DMFS between the two groups. Based on the survival data of the pure IMPC and IBC-NST cases claudin-4 positive tumors were associated with significantly shorter DMFS, suggesting a role of claudin-4 in cancer progression. If inverted polarity is a feature seen only in cancer cells, then better understanding of its development may provide crucial targets for therapy.

Further research will be necessary to understand the significance of the dysregulated polarity in IMPC cells and of the clustered tumor cells situated within empty stromal spaces.

## Data Availability

The original contributions presented in the study are included in the article/supplementary material, further inquiries can be directed to the corresponding author.

## Abbreviations

IMPC, Invasive micropapillary carcinoma; IBC-NST, Invasive breast carcinoma of no special type; DMFS, Distant metastasis free survival; OS, Overall survival; HR, Hazard ratio value; EMA, Epithelial membrane antigen; JAM-A, junctional adhesion molecule A; ZO-1, ZO-2, ZO-3, Zonula occludens-1, -2 and -3; DCIS, Ductal carcinoma *in situ*; *CLDN1, -3, -4, -7*, claudin-1, -3, -4 and -7 genes; LUM-A, Luminal-A; LUM-B1, Luminal B-HER2 negative; LUM-B2, Luminal B-HER2 positive; TNBC, Triple negative breast carcinoma; HR+, Hormone receptor positive; HR-, Hormone receptor negative; FFPE, Formalin fixed paraffin embedded; TJ, Tight junction; TGF-ß, Transforming growth factor-ß; ER, Estrogen receptor.
